# Elevated ubiquitination contributes to protective immunity against severe SARS‐CoV‐2 infection

**DOI:** 10.1002/ctm2.1103

**Published:** 2022-11-29

**Authors:** Yinggang Che, Dongbo Jiang, Yong Zhang, Junqi Zhang, Tianqi Xu, Yuanjie Sun, Jiangjiang Fan, Jiawei Wang, Ning Chang, Yingtong Wu, Shuya Yang, Leidi Xu, Jiaqi Ding, Chenchen Hu, Yinan Huang, Jian Zhang, Kun Yang

**Affiliations:** ^1^ Department of Immunology Basic Medicine School Air‐Force Medical University (The Fourth Military Medical University) Xi'an Shaanxi China; ^2^ Department of Respiratory Medicine Xijing Hospital Air‐Force Medical University (The Fourth Military Medical University) Xi'an Shaanxi China; ^3^ The Key Laboratory of Medicine for Bio‐Hazard Prevention and Treatment of People's Liberation Army Basic Medicine School, Air‐Force Medical University (The Fourth Military Medical University) Xi'an Shaanxi China; ^4^ Department of Thoracic Surgery Tangdu Hospital Air‐Force Medical University Xi'an Shaanxi China; ^5^ First Sanatorium of Air Force Healthcare Center for Special Services Hangzhou Zhejiang China; ^6^ Department of Rheumatology and Immunology Tangdu Hospital Air‐Force Medical University Xi'an Shaanxi China

**Keywords:** biomarker, immune system, prognosis, severe acute respiratory syndrome coronavirus 2, ubiquitination

## Abstract

**Background:**

The crosstalk between the ubiquitin‐proteasome and the immune system plays an important role in the health and pathogenesis of viral infection. However, there have been few studies of ubiquitin activation in severe acute respiratory syndrome coronavirus 2 (SARS‐CoV‐2) infection.

**Methods:**

We investigated the effect of ubiquitination on SARS‐CoV‐2 infection and patient prognosis by integrating published coronavirus disease 2019 (COVID‐19) multi‐transcriptome data and bioinformatics methods.

**Results:**

The differential expression of COVID‐19 samples revealed changed ubiquitination in most solid and hollow organs, and it was activated in lymphatic and other immune tissues. In addition, in the respiratory system of COVID‐19 patients, the immune response was mainly focused on the alveoli, and the expression of ubiquitination reflected increasing immune infiltration. Ubiquitination stratification could significantly differentiate patients' prognosis and inflammation levels through the general transcriptional analysis of the peripheral blood of patients with COVID‐19. Moreover, high ubiquitination levels were associated with a favourable prognosis, low inflammatory response, and reduced mechanical ventilation and intensive care unit. Moreover, high ubiquitination promoted a beneficial immune response while inhibiting immune damage. Finally, prognostic stratification and biomarker screening based on ubiquitination traits played an important role in clinical management and drug development.

**Conclusion:**

Ubiquitination characteristics provides new ideas for clinical intervention and prognostic guidance for COVID‐19 patients.

## Graphical abstract

During coronavirus disease 2019 infection, the ubiquitinated genes showed general downregulation in lung tissue and significant activation in the peripheral blood of patients. By ubiquitination expression profile stratification, patients with higher ubiquitination had a better prognosis and higher immune infiltration and response. The screening of biomarkers and the construction of the risk model present clinical application value for distinguishing critically ill patients.

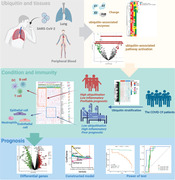

## 1 INTRODUCTION

Coronavirus disease 2019 (COVID‐19), caused by severe acute respiratory syndrome coronavirus 2 (SARS‐CoV‐2), has given rise to more than 524 million infections and more than 6.27 million deaths according to the statistics of the World Health Organization as of 22 May 2022. In addition, the variant of SARS‐CoV‐2 lineages including alpha, beta, gamma, and delta have contributed to deteriorated pandemic and threat to the public.^1^, ^2^, ^3^, ^4^ It is partly attributed to enhanced transmissibility and partial immune escape.^5^ Although most patients are asymptomatic or have mild symptoms, some patients progress to severe disease or die before long. It initially presents as a respiratory tract infection, including fever, dyspnoea, and myalgias, but it can rapidly progress to a more severe form.^6^ Severe COVID‐19 may affect multiple organ systems during the acute phase of the disease. The impairment of pulmonary, cardiac, and renal function and thromboembolism have been described in severely ill and deceased patients.^7^, ^8^ Patients who recover from mild to moderate COVID‐19 suffer from modest subclinical multi‐organ effects related to thrombotic, pulmonary, cardiac, and renal function.^9^ Therefore, it is crucial to understand the COVID‐19 mechanisms to control disease development and evaluate the patient's clinical prognosis.

Ubiquitination is a type of eukaryotic protein modification catalysed by a three‐enzyme cascade (E1 ubiquitin‐activating enzyme, E2 ubiquitin‐conjugating enzyme, and E3 ubiquitin ligase enzyme) and reversed by deubiquitinating enzymes.^10^ For protein ubiquitination modification, E3s usually determine substrate specificity, and E2s can play an important role in substrate selection. Ubiquitin and its modifier enzymes are involved in transcription, signal transduction, membrane trafficking, and innate and adaptive immune responses. Additionally, viruses are connected to ubiquitin and ubiquitin‐like modifiers in various ways. Many viruses encode proteins that alter substrate specificity to favour replication by modifying the host's ubiquitin and ubiquitin‐like machinery. Viruses can alter proteasome degradation machinery to interfere with class I major histocompatibility complex (MHC‐I)‐restricted antigen presentation. Viral proteins can be directly modified by ubiquitin or ubiquitin‐like proteins, and some viruses even encode their own ubiquitinating or deubiquitinating enzymes. Viruses, in turn, depend on the ubiquitination and degradation of host surface receptors as countermeasures to escape from T cell recognition.^11^ However, host cells utilise the ubiquitin‐proteasome system to counteract viral infections by generating target structures recognized by T cells.

Most viral proteins can be modified posttranslationally, and 21 of the 27 detected SARS coronavirus proteins were also ubiquitinated.^12^ Viruses commonly hijack ubiquitination pathways for replication and pathogenesis, impacting targeted protein degradation.^13^ The ubiquitin molecules of the host‐infected SARS‐CoV‐2 virus closely interact with the SARS‐CoV‐2 protein. ZYG11B, serving as a substrate adapter subunit in the E3 ubiquitin ligase complex ZYG11B‐CUL2‐Elongin BC, may bind to the N‐terminal glycine in ORF10, SARS‐CoV‐2 protein, to target it for degradation, and ORF3 interacts with TRIM47(E3) to impact ubiquitin‐like ligase activity.^14^, ^15^ In addition, many ubiquitin sites are differentially expressed after infection with SARS‐CoV‐2.^12^ Hence, ubiquitination plays a crucial role in patients infected with SARS‐CoV‐2, indicating that these acquired adaptations can be posttranslationally modified and may recruit cellular proteins with specific functions. Ubiquitin is also associated with the immune response to SARS‐CoV‐2. When the SARS‐CoV‐2 N protein is ectopically expressed, innate immune RIG‐I activation is decreased as a result of the antagonistic activity of TRIM25.^16^

In our research, we integrated multiple published public datasets to investigate the biological characteristics in the ubiquitination of COVID‐19 patients (Table S1). The ubiquitin gene profile (Table S2) first demonstrated the specific biological characteristics and clinical prognosis of COVID‐19 patients in multiple organs. After infection with SARS‐CoV‐2, ubiquitin gene expression changed apparently in the autopsy of the lung as a suffering organ. The change in ubiquitin expression in A549 cells transfected with SARS‐CoV‐2 was verified in a time‐dependent manner. Additionally, ubiquitin expression was higher in the immune histology after SARS‐CoV‐2 infection and was associated with the immune response. In blood leukocytes of COVID‐19 patients, the ubiquitin‐associated pathway was significantly upregulated. Based on ubiquitin subclustering, the high ubiquitin cluster was correlated with a lower inflammatory reaction and a favourable prognosis in COVID‐19 patients, presenting higher immune infiltration and response. As a result, higher ubiquitin levels could be a protective factor in COVID‐19 patients. Based on prognostic stratification, constructing a risk model and screening biomarkers could shed novel light on promising clinical intervention and management.

## 2 RESULTS

### 2.1 The profile of ubiquitin gene expression and dynamic changes in SARS‐CoV‐2 infection

**FIGURE 1:**
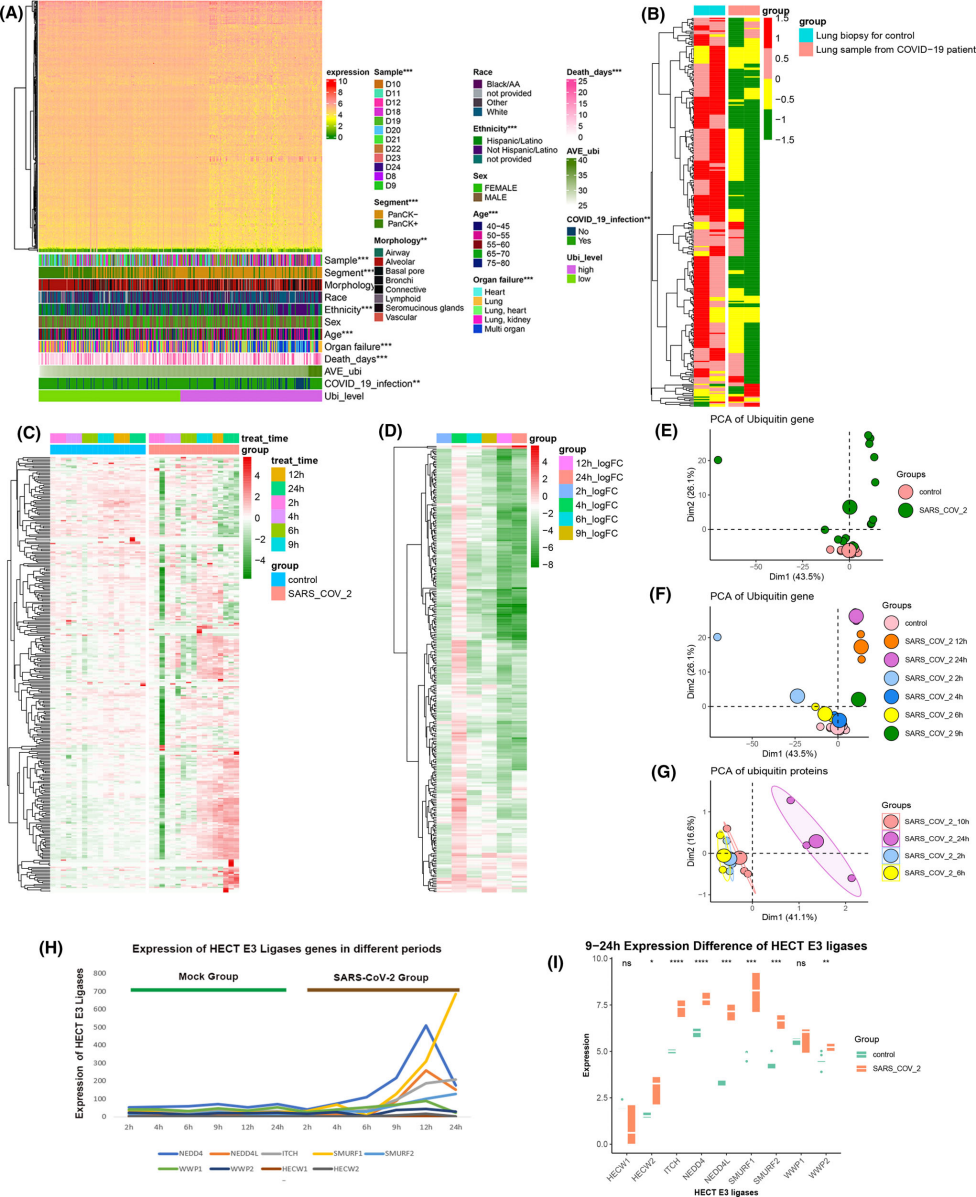
After infection with coronavirus disease 2019 (COVID‐19, ubiquitin‐related genes significantly change in lung tissue. (A) The comprehensive heatmap demonstrated the expression of ubiquitin‐enzyme genes in the samples originating from multiple organs. Additional annotation of the abscissa included samples, segment, morphology, race, ethnicity, sex, age, organ failure, death days, average ubiquitin expression (AVE Ubi), COVID‐19 infection, and ubiquitin level (Ubi level). The ubiquitin level was compared with other factors calculated with the chi‐square test. (B) The heatmap (z‐scored by row) demonstrates the differential expression of ubiquitin genes between lung biopsies from healthy negative controls and postmortem COVID‐19 patients. (C) The heatmap (z‐scored by row) shows the distinct expression of ubiquitin genes in A549 cells at different periods (2, 4, 6, 9, 12, and 24) between control and severe acute respiratory syndrome coronavirus 2 (SARS‐CoV‐2)‐infected cells. Additional annotation of the abscissa included A549 cell treatment time and grouping control and SARS‐CoV‐2. (D) Heatmaps (z‐scored by row) of log2‐fold changes in ubiquitin genes in the COVID‐19 infection and control groups at different periods (2, 4, 6, 9, 12, and 24, also see Table S4). (E) The principal component analysis (PCA) plot of ubiquitin genes was divided into control and SARS‐CoV‐2 groups in the GSE184536 dataset. (F) The PCA plot of ubiquitin genes was grouped into the control and SARS‐CoV‐2 groups at different periods of the GSE184536 dataset (2, 4, 6, 9, 12, and 24). (G) The PCA plot of ubiquitin proteins was annotated by SARS‐CoV‐2 of the different periods of the PXD017710 dataset (2, 6, 10, and 24). (H) Expression changes of HECT E3 ligase family genes in mock and infection groups at different times of infection (2, 4, 6, 9, 12, and 24 h). Green represents the mock group, and brown represents the SARS‐CoV‐2‐infected group. (I) Boxplot shows the expression differences of HECT E3 ligase family genes after 9–24 h SARS‐CoV‐2‐infected and mock groups. (****: *p* < .0001, ***: *p* < .001, **: *p* < .01, *: <.05, ns: not significant)

By calculating the mean of ubiquitin enzyme genes in autopsy donors, we found that it was closely related to donor sample, ethnicity, age, cell segment, days from symptom onset to death, morphology, organ failure, and COVID‐19 infections (Figure 1A). The samples originating from donors varied in ubiquitin expression (Figure S1A), which decreased with age (Figure S1B). However, it was not differentially expressed in sex (Figure S1C). Notably, ubiquitin in the race showed a difference in characteristics, and the ethnicity of Hispanic/Latino was associated with low expression of ubiquitin (Figure S1D‐E). The patients had differential ubiquitin expression in multiple organ failure (Figure S1F). In general, SARS‐CoV‐2 infection was negatively correlated with ubiquitin expression, which decreased in COVID‐19 donors (Figure S1G). This result indicated that ubiquitination could be impacted by SARS‐CoV‐2 infection.

To illuminate the potential relationship between ubiquitination and SARS‐CoV‐2, we collected autopsy lung tissue from COVID‐19 patients and healthy donors. The heatmap demonstrated that the expression of COVID‐19 patients' ubiquitin genes was significantly different compared to the healthy donor (Figure 1B). To address the ubiquitin gene characteristics of lung tissue infected with SARS‐CoV‐2, we analysed ubiquitin gene changes in different periods (2, 4, 6, 9, 12, and 24 h) in A549 cells infected with SARS‐CoV‐2 and controls (Figure 1C and Table S3). In the early 2–6 h, the ubiquitin genes were consistent with the control and SARS‐CoV‐2 groups. Starting with 9 h of infection with SARS‐CoV‐2, the group infected with SARS‐CoV‐2 significantly differed from the control groups. Differential expression analyses of ubiquitin genes of control and infection in different periods changed over the infection time from 9 to 24 h (Figure 1D and Table S4). Additionally, based on the principal component analysis (PCA) of ubiquitin genes, the cells infected for 2, 4, and 6 h were not different from the control group. The cells infected for 9, 12, and 24 h showed an apparent disparity. With a longer time of infection, the distinction of ubiquitin genes was more apparent (Figure 1E,F). This phenomenon was verified by ubiquitin‐protein expression in Caco‐2 cells (Figure 1G). As Novelli et al. mentioned,^17^ the HECT E3 family was significantly elevated after COVID‐19 infection, consistent with our study, especially 9 h after COVID‐19 infection (Figure 1H,I). Therefore, after infecting SARS‐CoV‐2, ubiquitin gene expression presented a distinctly time‐dependent change in the lung, which is a sensitive organ for SARS‐CoV‐2 infection.

### 2.2 Ubiquitin expression was closely associated with the immune landscape in COVID‐19 patients

**FIGURE 2:**
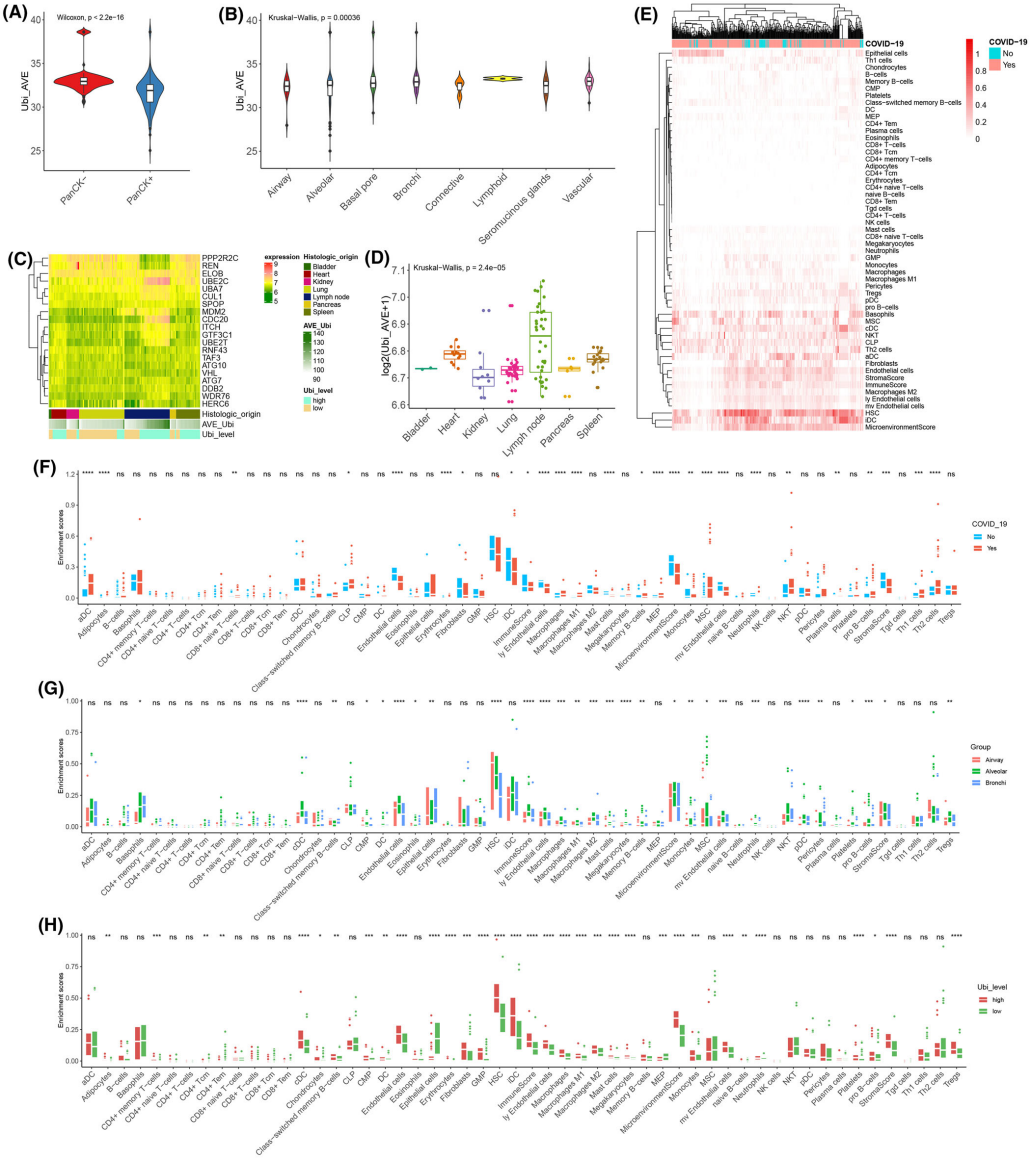
Interaction between ubiquitin and the immune system after coronavirus disease 2019 (COVID‐19) infection. (A) The violin plot shows the difference in average ubiquitin expression in the PanCK +/‐groups by the Wilcoxon test (*p* < 2.2e‐16). (B) The violin plot shows the difference in average ubiquitin expression in the different morphologies by the Kruskal–Wallis test (*p* = .004). (C) The comprehensive heatmap of partial ubiquitin genes in distinct tissues. Additional annotations of the abscissa included histologic origin, AVE‐Ubi, and Ubi level. (D) The boxplot plot shows the difference in average ubiquitin expression in the different histologies by the Kruskal–Wallis test (*p* = 2.4e‐05). (E) The heatmap (z‐scored by row) shows the enrichment score of the different cells in the non‐COVID‐19 and COVID‐19 groups. (F) The boxplot plot shows the difference in various cells in the non‐COVID‐19 and COVID‐19 groups by *t*‐test. (G) The boxplot plot shows the difference in various cells in the alveolar, airway, and bronchi by the Kruskal–Wallis test. (G) The boxplot plot shows the difference in various cells in the alveoli's high and low ubiquitin groups by *t*‐test. (****: *p* < .0001, ***: *p* < .001, **: *p* < .01, *: <.05, ns: not significant)

As mentioned, the expression of ubiquitin genes varies from tissue to tissue. Consistently, the phenomenon was observed in COVID‐19 patients. As epithelial cell biomarkers, pan‐cytokeratin positive cells demonstrated lower ubiquitin gene expression and low ubiquitination activity (Figure 2A). Analysing the differential ubiquitin level in the distinct morphology, the expression of ubiquitin genes was the highest level of lymphoid cells in the COVID‐19 patients (Figure 2B). It is well known that the immune system rapidly responds to the invading SARS‐CoV‐2 virus, and high‐level ubiquitin expression of lymphoid morphology potentially correlates with the immune response. Another transcriptomic data of COVID‐19 patients' autopsy manifested the phenomenon in which the highest ubiquitin expression occurred in the lymphoid, although only 20 ubiquitin genes were involved (Figure 2C,D).

Subsequently, we analysed the immune landscape of non‐COVID‐19 and COVID‐19 donors (Figure 2E). In the autopsy donor, the death infection SARS‐CoV‐2 demonstrated a low immune response and immune profiles, which showed that the death caused by COVID‐19 patients was attributed to the descending immune state, concomitant with low immune cell infiltration, low immune scores, and low microenvironments (Figure 2F).

As a common injury location of SARS‐CoV‐2 infection, we profiled the immune landscape of the respiratory system. Compared with the airway and bronchi of COVID‐19 patients, the alveoli demonstrated the highest immune cell infiltration and responses (Figure 2G). In the alveoli, consistent with the total immune trait, the death infection SARS‐CoV‐2 was associated with lower immune profiles than the control (Figure S1H). For the alveoli of COVID‐19 patients, the ubiquitin level was closely associated with the immune landscape. The higher expression of ubiquitination manifested in a higher immune response and infiltration (Figure 2H). Hence, ubiquitination reflected the immune cell infiltration and response and was positively correlated with it.

### 2.3 The ubiquitin‐associated pathway profiles in blood leukocytes of COVID‐19 patients

**FIGURE 3:**
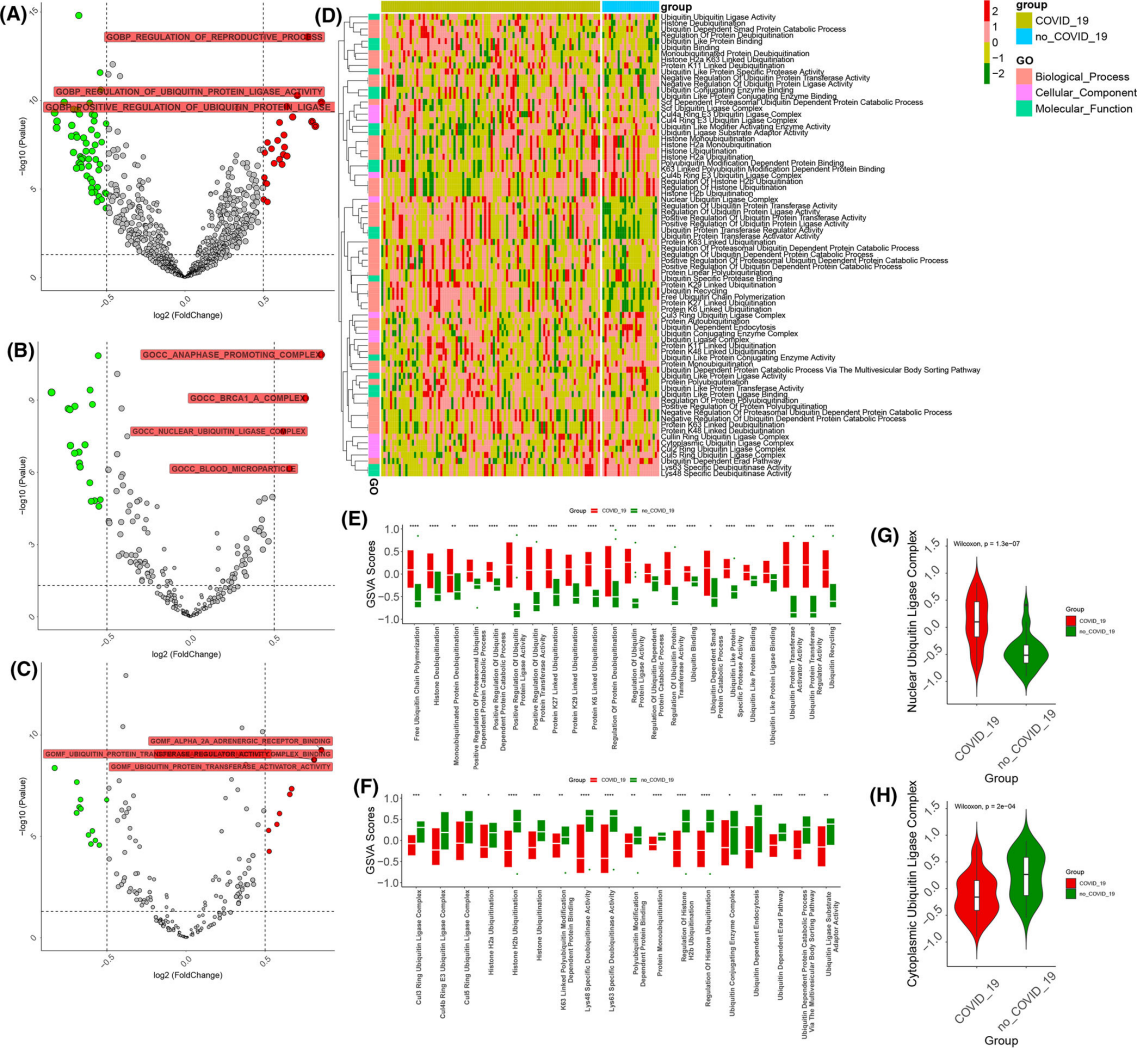
Ubiquitin pathway profiles of peripheral blood mononuclear cells in coronavirus disease 2019 (COVID‐19) patients. (A–C) The volcano plot shows the up‐ and downregulated biological processes, cellular components, and molecular functions in the blood leukocytes of COVID‐19 and non‐COVID‐19 patients. Red: upregulation, green: downregulation. (D) The heatmap (z‐scored by row) shows all ubiquitin‐associated pathways in the blood leukocytes of COVID‐19 and non‐COVID‐19 patients. The annotation of the vertical axis is the biological procession, cellular component, and molecular function. (E,F) The boxplot plot shows the up‐ and downregulated ubiquitin‐associated pathways in the blood leukocytes of COVID‐19 patients by *t*‐test. (G) The violin plot shows the difference in the nuclear ubiquitin ligase complex in the blood leukocytes of COVID‐19 and non‐COVID‐19 patients by the Wilcoxon test (*p* = 1.4e‐0.7). (H) The violin plot shows the difference in the cytoplasmic ubiquitin ligase complex in the blood leukocytes of COVID‐19 and non‐COVID‐19 patients by the Wilcoxon test (*p* = .00024). (****: *p* < .0001, ***: *p* < .001, **: *p* < .01, *: <.05)

Based on the crosstalk between ubiquitination and immune profiles infecting SARS‐CoV‐2, we analysed their association in peripheral blood leukocytes. By annotating the Gene Ontology enrichment pathway, gene set variant analysis (GSVA) illuminated the ubiquitin characteristics at the pathway level in peripheral blood leukocytes. We acquired 1944 biological process pathways, 309 cellular component pathways, and 287 molecular function pathways based on the ubiquitin genes of GSVA. By differential analysis of non‐COVID‐19 and COVID‐19 patients, we found that 47, 4, and 11 pathways were upregulated, and 192, 32, and 21 were downregulated in the BP, CC, and MF pathways, respectively (Figure 3A–C). Compared with the non‐COVID‐19 patients, there were higher enrichment scores in the COVID‐19 patients for regulation of ubiquitin‐protein ligase activity, positive regulation of ubiquitin‐protein ligase activity, nuclear ubiquitin ligase complex, ubiquitin‐protein transferase activator activity, ubiquitin‐protein transferase regulator activity, and ubiquitin‐like protein‐specific protease activity. As a result, ubiquitin has a close relationship with patients infected with COVID‐19, and partial ubiquitin‐associated pathways were more highly expressed in the blood leukocytes of COVID‐19 patients. Wang et al. found the ubiquitin‐dependent protein catabolic process upregulated in the COVID‐19 group.^18^ We summarised the all‐ubiquitin pathways of GSVA and observed the dynamic characteristics of ubiquitin pathways in the non‐COVID‐19 and COVID‐19 patients (Figure 3D). Free ubiquitin chain polymerisation and protein K6‐ K27‐, and K29‐linked ubiquitination pathways demonstrated higher activity in COVID‐19 patients (Figure 3E). It has been reported that the K6 chain of ubiquitin is associated with DNA damage,^19^ the K27 ubiquitin chain acts in innate immunity, protein homeostasis, and DNA damage,^20^, ^21^, ^22^ and the K29 ubiquitin chain is related to innate antiviral immunity.^23^

Nevertheless, histone H2B ubiquitination and the CUL3, CUL4A, and CUL5 ring ubiquitin ligase complex and LYS63‐specific deubiquitinate activity pathways were higher in the non‐COVID‐19 patients (Figure 3F). Cullin ring ubiquitin ligases CUL3, CUL4A, and CUL5 are related to DNA repair and ROS regulation, controlling DNA repair and replication, and the immune system.^24^ Hence, SARS‐CoV‐2 contributes to DNA damage and inhibits DNA repair and replication. Furthermore, compared with non‐COVID‐19 patients, the ubiquitin ligase complex was more active in the nucleus and less active in the cytoplasm in COVID‐19 patients, which indicated that ubiquitin‐associated functions were focused on the nuclear region (Figure 3G,H). It is attributed partly to the important contribution of the ubiquitin‐proteasome system to DNA damage and impairment, or misfolded proteins in the nucleus are a greater threat than in other compartments.^25^, ^26^ These results demonstrated ubiquitin pathways associated with SARS‐CoV‐2 infection and differential nuclear and cytoplasmic traits.

### 2.4 High levels of ubiquitination were closely associated with a lower inflammatory response and better clinical outcomes in the peripheral blood leukocytes of COVID‐19 patients

**FIGURE 4:**
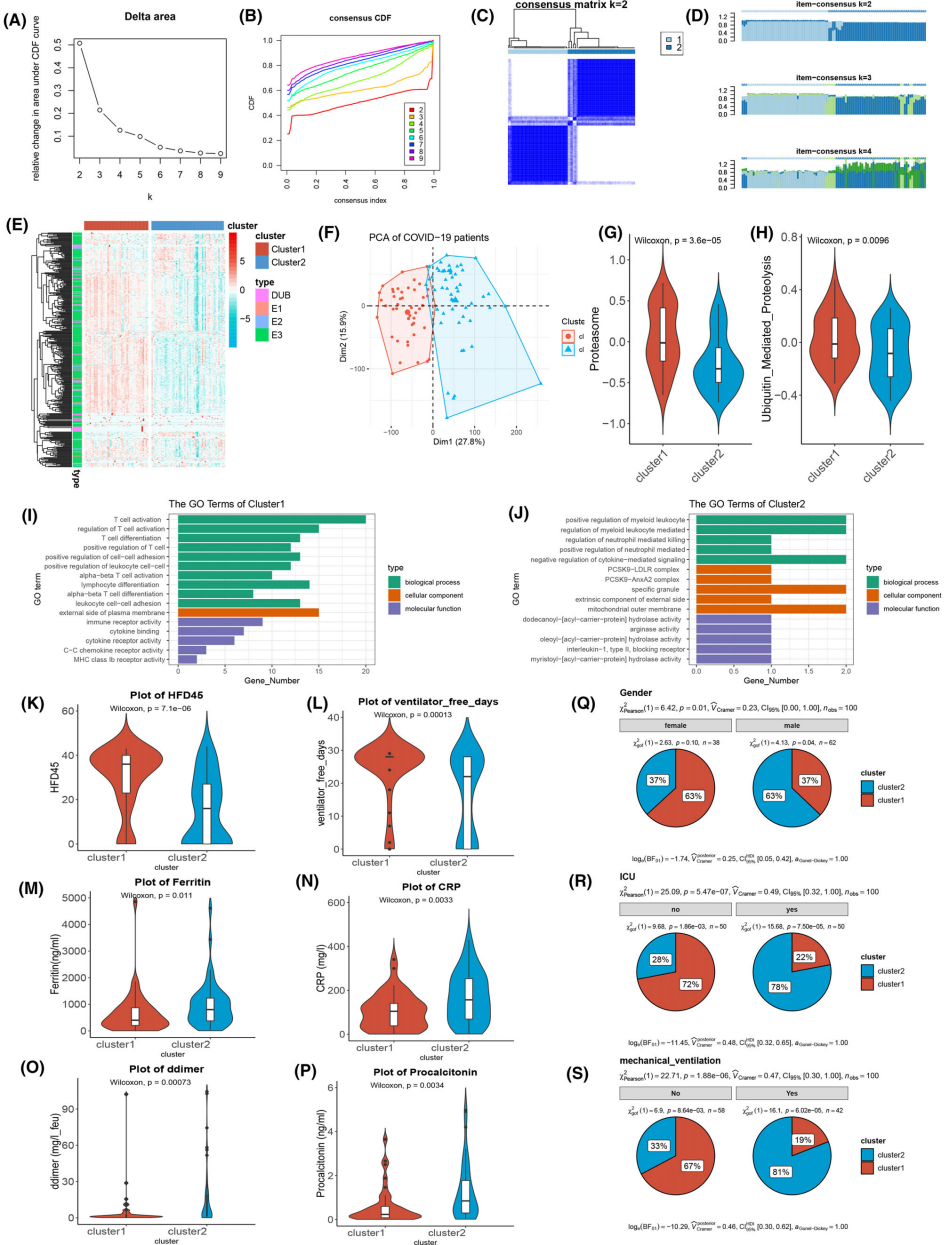
A close association between ubiquitination stratification and the clinical outcome of patients in PBMC. (A,B) The delta area plot and consensus consistent cumulative distribution function (CDF) plot of analysis ubiquitin genes by consensus clustering in the blood leukocytes of coronavirus disease 2019 (COVID‐19). (C,D) The cluster heatmap and item consensus plots of the analysis of ubiquitin genes by consensus clustering in the blood leukocytes of COVID‐19 patients. (E) The heatmap (z‐scored by row) shows the ubiquitin gene expression of subclusters and high expression in cluster 1. The vertical axis annotated the E1s, E2s, E3s, and DUBs. (F) The principal component analysis plot of all transcriptome genes was grouped by clusters 1 and 2 in the blood leukocytes of COVID‐19 patients. (G) The violin plot shows that proteasome and ubiquitin‐mediated proteolysis were more active in the blood leukocytes of COVID‐19 tested by the Wilcoxon test. (I,J) Bar plot of GO enrichment analysis of cluster1 upregulating genes and cluster2 upregulating genes. (K–P) The violin plot shows the difference, including hospital‐free day, free ventilator days, ferritin, C‐reactive protein (CRP), D‐dimer, and procalcitonin, in clusters 1 and 2. (Q–S) The pie plot shows the proportion of males and females, ICU, and mechanical ventilators in clusters 1 and 2

Regarding ubiquitin's importance in the peripheral blood leukocytes of COVID‐19 patients, we performed consensus clustering to divide the appropriate subgroups to study potential biological functions and clinical indicators. According to the delta area plot and consensus consistent cumulative distribution function , the COVID‐19 patients reasonably subclassed two clusters based on the ubiquitin genes (Figure 4A,B). The cluster heatmap and item consensus plots demonstrated the stability of the two clusters (Figure 4C,D). Ubiquitin genes were highly expressed in cluster 1 but reversely expressed in cluster 2 (Figure 4E). Additionally, there was an excellent distinguishing capacity of ubiquitin subclusters in the all‐transcript genes (Figure 4F). At the pathway level, we analysed the difference between clusters 1 and 2 through GSVA annotating Kyoto Encyclopedia of Genes and Genomes pathways. Proteasome‐ and ubiquitin‐mediated proteolysis were more highly enriched in cluster 1 (Figure 4G,H). Therefore, cluster 1 expressed highly ubiquitin genes and higher activity of ubiquitin function.

By analysing clusters 1 and 2 differences in transcript genes, these genes were subjected to gene ontology (GO) pathway enrichment. Cluster 1 was enriched in the T cell activation‐associated pathway, C–C chemokine receptor and MHC class II receptor activity‐associated pathways, and other immune‐associated pathways (Figure 4I). Positive regulation of myeloid leukocytes, negative regulation of cytokine‐mediated signalling, and regulation of neutrophil‐mediated killing, were enriched in cluster 2 (Figure 4J). This finding illuminated the difference in the immune response and pathways of the ubiquitin subclusters in COVID‐19 patients.

In addition, ubiquitin subclusters distinguished the inflammatory response and clinical prognosis of COVID‐19 patients (the clinical information of the patients are shown in Table S5). As an indicator reflecting the prognosis of patients, hospital‐free days at day 45 (HFD‐45) assign a zero value (0‐free days) to patients who remain admitted longer than 45 days or die, while admitted and higher values of HFD‐45 assign a zero value to patients with shorter hospitalisations and milder disease severity.^27^ The patients in cluster 1 had higher scores on the HFD‐45 than those in cluster 2, showing milder disease severity in cluster 1 (Figure 4K). The free ventilator days of cluster 1 were also lengthened compared with those of cluster 2 of COVID‐19 patients, which showed that the high expression of ubiquitin genes was a protective prognostic indicator (Figure 4L). As clinical inflammatory indicators, ferritin, C‐reactive protein, D‐dimer, and procalcitonin increased in cluster 2, representing an elevated inflammatory reaction (Figure 4M–P). D‐dimer and inflammatory markers (including hsCRP and ferritin) were significantly higher in severe cases than in moderate cases.^28^ Additionally, there were more patients in the male, intensive care unit (ICU), and mechanical ventilator groups in cluster 2 than in cluster 1, confirming that cluster 2 had a higher risk and poor prognosis (Figure 4Q–S). The outcomes showed that COVID‐19 patients' lower ubiquitin gene expression had a poor prognosis and higher inflammation, negatively associated with the immune response.

### 2.5 The immune infiltrating characteristics of ubiquitin subclusters in COVID‐19 patients

**FIGURE 5:**
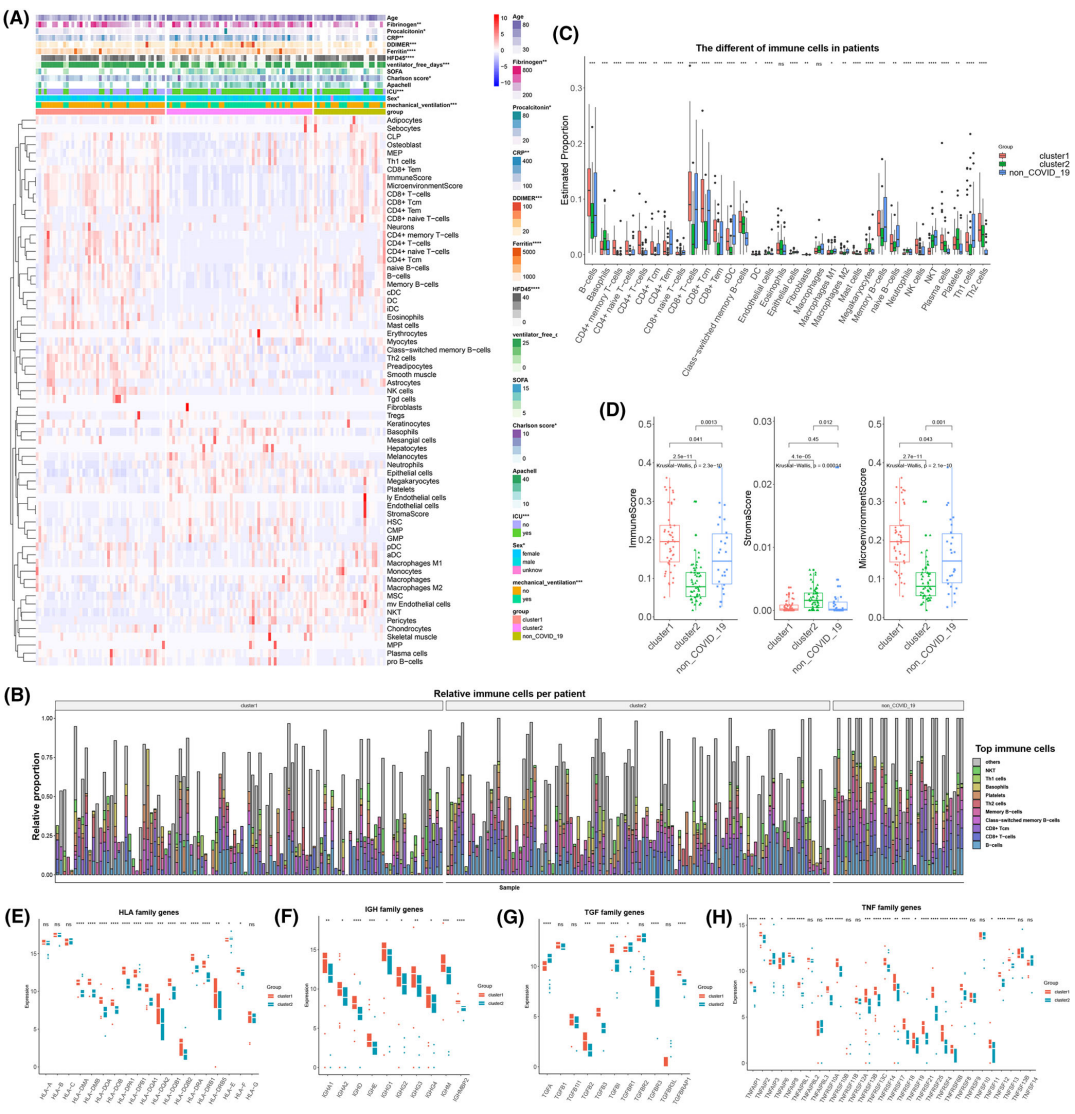
The immune profile of ubiquitin stratification in PBMCs. (A) The comprehensive heatmap demonstrates the enrichment scores of various cells in cluster 1, cluster 2, and the non‐coronavirus disease 2019 (non‐COVID‐19) group. Additional annotation of the abscissa included group, mechanical ventilation, sex, intensive care unit, acute physiology and chronic health evaluation (APACHE‐II) scores, Charlson score, sequential organ failure assessment score, ventilator‐free days, hospital‐free days at day 45, ferritin, D‐dimer, CRP, procalcitonin, fibrinogen, and age. Categorical variables are compared with other factors using the chi‐square test, and continuous variables are compared with the Kruskal–Willis test. (B) The proportion of the top 10 immune cells in each sample of cluster 1, cluster 2, and non‐COVID‐19 patients. (C) The boxplot shows the difference between the various cells in clusters 1 and 2 and the non‐COVID‐19 patients. (D) The boxplot shows the difference in immune score, stromal score, and microenvironment in cluster 1, cluster 2, and the non‐COVID‐19 group. (E–H) The boxplot shows the difference between the human leukocyte antigen family, immunoglobulin heavy chain (IGH) family, transforming growth factor family, and tumour necrosis factor family in clusters 1 and 2 of COVID‐19 patients. (****: *p* < .0001, ***: *p* < .001, **: *p* < .01, *: <.05, ns: no significance)

The prognosis and inflammation of ubiquitin subcluster patients were closely related to the different immune response patterns and cells. The immune infiltrating cells and clinical indicators differed in cluster 1, cluster 2, and non‐COVID‐19 patients and reflected distinct response traits (Figure 5A,B and Figure S2A–D). Th1 cells, which are essential for host defence against intracellular pathogens, were decreased in patients, but Th2 cells were increased in COVID‐19, and the Th1/Th2 balance in COVID‐19 has been associated with the outcome of the disease^29^, ^30^ (Figure 5C). Compared with COVID‐19 patients, plasma cells and platelets were lower in non‐COVID‐19 patients, showing that platelets were upregulated and humoral immunity was active after infection with SARS‐CoV‐2 (Figure 5C). COVID‐19 patients in cluster 2 had fewer infiltrating B cells, T cells, and natural killer (NK) cells, which was consistent with the fact that severe forms of COVID‐19 are characterised by a marked decrease in the total number of peripheral blood lymphocytes, including both CD4+ and CD8+ T cells, B lymphocytes, and NK cells^31^ (Figure 5C). Nevertheless, neutrophils, megakaryocytes, and NKT cells were significantly increased in cluster 2 (Figure 5C). Neutrophils and monocytes were described as an indicator of severe respiratory symptoms and poor prognosis in patients with COVID‐19,^32^ probably attributed to the increased formation of neutrophil extracellular traps.^33^

Furthermore, the immune and microenvironment scores were higher in cluster 1, and the stromal score was increased in cluster 2, representing the disparity in immune response traits of ubiquitin subclusters (Figure 5D). Higher ubiquitin expression, as a protective factor, was related to the active immune response in the peripheral blood leukocytes of COVID‐19 patients.

Immune‐associated molecular genes are expressed differently in the subgroups and interact with immune cells. Many MHC II molecules, immunoglobulin heavy locus (IGH), transforming growth factor (TGF), tumour necrosis factor (TNF), and interleukin (IL) family members were enriched in cluster 1 and improved the immune response. Partial genes of the cytokine storm (IL1R1, IL1R2, ILRL1, IL1RL2, INFAR1, INFGR1, INFGR2, and IFNK) were overexpressed in cluster 2, illuminating the poor prognosis (Figure 5E–H and Figure S2E,F). Hence, a complex and comprehensive interaction of immune cells and cytokines gives rise to patients' clinical outcomes in COVID‐19 infection. Upregulated ubiquitin expression could harvest the beneficial immune response and prevent poor prognosis.

### 2.6 Construction of a protective model and screening biomarker for COVID‐19 patients

**FIGURE 6:**
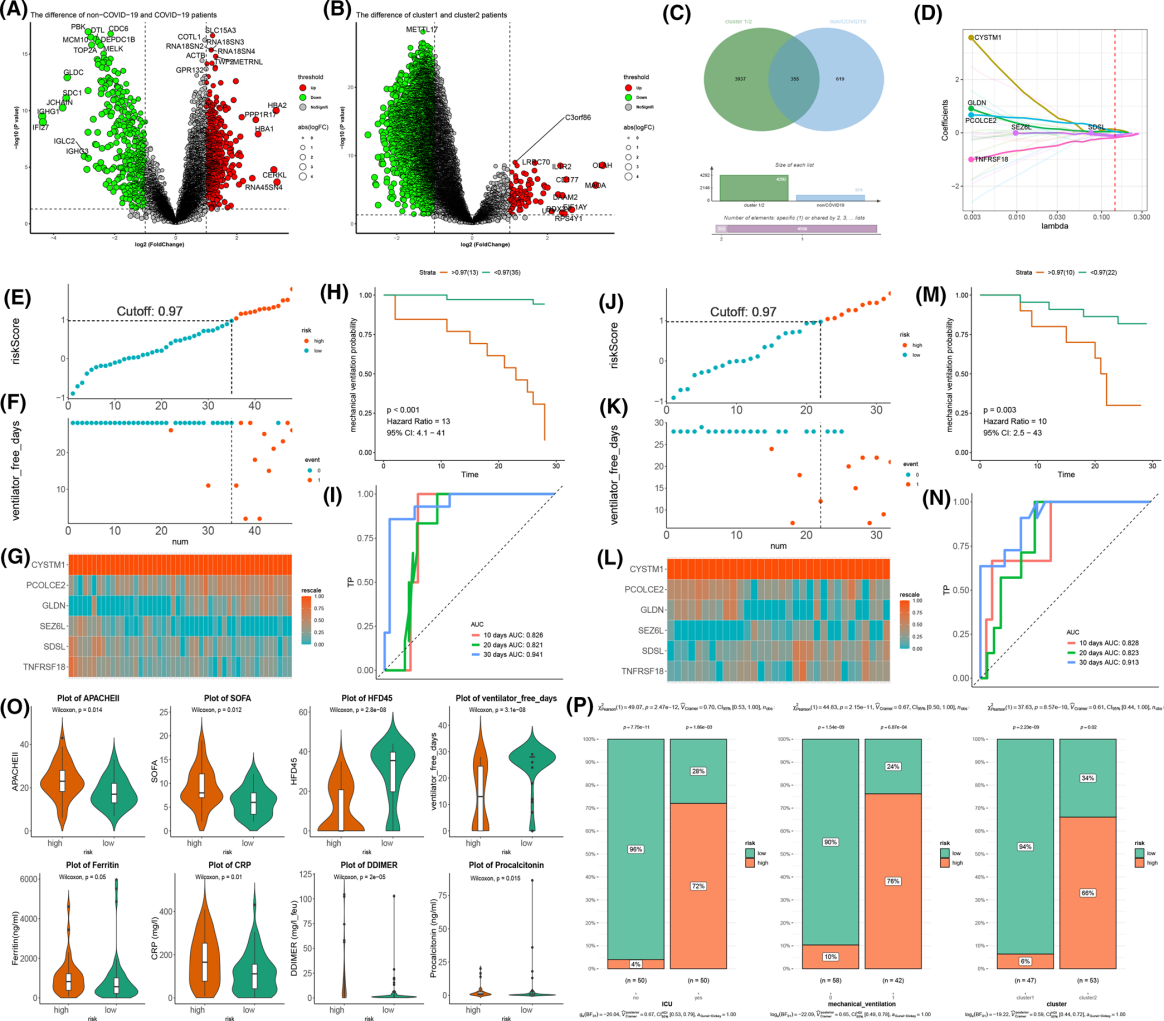
Construction of prognostic models and screening of biomarkers for coronavirus disease 2019 (COVID‐19) patients. (A,B) The volcano plot shows the u‐p and downregulated genes in the blood leukocytes of non/COVID‐19 and cluster 1/2 patients. Red: upregulation, green: downregulation. (C) Venn diagram shows that 355 genes were screened by intersecting differentially expressed genes between cluster 1/2 and non/COVID‐19 patients. (D) Six genes are selected to construct the risk model by least absolute shrinkage and selection operator regression. (E) The dot plot shows the patients' split between the high‐ and low‐risk groups according to the 0.97 cutoff value in the training data. (F) The scatter plot shows the relation between the risk score and ventilator‐free days of the risk model in the training data. 0, no mechanical ventilator; 1, mechanical ventilator. (G) Heatmap of the expression of six genes in the training data. (H) The training data shows the mechanical ventilation probability plot of the high‐ and low‐risk groups. (I) 10‐, 20‐, and 30‐day ROC curves of the risk model in the training data. (J) The dot plot shows the patients' split between the high‐ and low‐risk groups according to the 0.97 cutoff value in the testing data. (K) The scatter plot shows the relation between the risk score and ventilator‐free days of the risk model in the testing data. 0, no mechanical ventilator; 1, mechanical ventilator. (L) Heatmap of the expression of six genes in the testing data. (M) The testing data shows the mechanical ventilation probability plot of the high‐ and low‐risk groups. (N) 10‐, 20‐, and 30‐day ROC curves of the risk model in the testing data. (O) The violin plot shows the difference, including APACHE‐II score, sequential organ failure assessment score, hospital‐free day, free ventilator days, ferritin, CRP, D‐dimer, and procalcitonin, in the high‐ and low‐risk groups. (P) The bar chart shows the proportion of ICU, mechanical ventilators, and ubiquitin subclusters

To seek a biomarker reflecting the clinical prognosis in peripheral blood leukocytes of COVID‐19 patients, we differentially analysed the transcripts of non‐COVID‐19 and COVID‐19 patients, screening the 974 differentially expressed genes (Figure 6A). Meanwhile, to reflect the severity of COVID‐19 patients, differential analysis of clusters 1 and 2 transcript data was performed to screen 4292 genes (Figure 6B). Intersecting different genes of non/COVID‐19 and cluster 1/2 patients, we screened 355 same genes to construct the risk model and biomarker genes (Figure 6C). Through the constructed model in the training set (including 48 patients) by least absolute shrinkage and selection operator (LASSO) regression, 6 crucial genes were screened to construct the prognostic model (Figure 6D). According to the cutoff value equalling 0.97 of the risk models, the patients were classified into high‐ and low‐risk groups (Figure 6E–G). There was a statistical significance for the mechanical ventilation probability curve in the groups (*p* < .05) (Figure 6H). The receiver operating characteristic curves (ROC) curve's 10‐, 20‐, and 30‐day areas were 0.826, 0.821, and 0.941, respectively, which showed that the ubiquitin model had good predictive capacity (Figure 6I). In the test data (including 32 patients), the ubiquitin model demonstrated the stability of COVID‐19 patients according to the cutoff value of 0.97 (Figure 6J–L). The mechanical ventilator probability was statistically significant in the high‐ and low‐risk groups (*p* < .05), and the 10‐, 20‐, and 30‐day areas under the ROC curve were 0.828, 0.823, and 0.913, respectively, in the test data (Figure 6M.N).

As indicators evaluating disease severity and organ failure, acute physiology and chronic health evaluation (APACHE II) and sequential organ failure assessment (SOFA) scores were elevated in the high‐risk group. A high‐risk group of patients consistently brought down their HFD‐45 score and ventilator‐free days. Additionally, the inflammatory response heightens in the high‐risk group, escalating in the ferritin, CRP, D‐dimer, and procalcitonin, inversely in the low‐risk group (Figure 6O). ICU and mechanical ventilator were enriched in the high‐risk group, showing the severity of patients. Regarding ubiquitin subclusters, the low‐risk group was closely associated with cluster 1, and cluster 2 was concentrated in the high‐risk group, consistent with the analysis above (Figure 6P). The risk model was fairly predictive and capable of patients' clinical prognostic and inflammatory responses. Six biomarkers of the model (CYSTM1, PCOLCE2, GLDN, SEZ6L, SDSL, and TNFRSF18) demonstrated the differential expression of COVID‐19′s prognostic and shed light on the clinical biomarkers to apply to patients (Figure S3A and Table S6). Sixty‐seven molecules correlated with marker genes were screened through protein–protein networks (Figure S3B). These molecules were associated with collagen fibril organisation, amino acid biosynthetic and metabolic processes, and Treg regulation by GO enrichment analysis, which reflected the prognostic value of COVID‐19 patients (Figure S3C).

## 3 DISCUSSION

Ubiquitination levels are affected by various factors and differ after infection by SARS‐CoV‐2. The contribution of gross pathology to the rapid understanding of these two factors has provided a bridge to the understanding of ubiquitination and COVID‐19 infection through the multi‐omics study of these autopsy samples. Our study revealed that multiple factors were associated with the expression of ubiquitin‐associated genes by indiscriminately analysing ubiquitination levels in multiple tissues and organs. The development of diseases involving ubiquitination was analysed comprehensively by biocomputation and immunological estimation.

Ubiquitination is crucial for antiviral defence, and viral proteins can also be modified by ubiquitination.^34^ As the affected target, lung tissues exhibited a significant change in the expression of ubiquitination‐related enzymes after SARS‐CoV‐2 infection, and cytological verification showed a time‐dependent effect. Novelli et al. found that HECT E3 ligase is overexpressed after infection with SARS‐CoV‐2, which is consistent with our results, and inhibition of HECT E3 ligases as a potential therapy for COVID‐19.^17^ Eleven SARS‐CoV‐2 proteins contain 135 ubiquitin residues, from at least 1 ubiquitination residue to 66 residues of a single protein.^35^ It has been reported that the deubiquitinase USP29 promotes SARS‐CoV‐2 virulence by preventing proteasome degradation of ORF9b.^36^ Strikingly, compared to the lower ubiquitination expression in other tissues, the lymphatic tissue possessed activated ubiquitination, probably relating to the host immune response against SARS‐CoV‐2.

Meanwhile, Estimation of STromal and Immune cells in MAlignant Tumour tissues using expression data determined that immune cells and response were more focused on the end‐stage lung units, alveolar, than the airway and bronchi. As the airway gradually expands, immune infiltration gradually increases over the terminal lung; for instance, the infiltration of macrophages, memory B cells, and immune scores increase and are associated with high ubiquitination. Mucociliary clearance biases infectious virion and infected cells to spread in the alveolar region of the deep lung, where the infection arises from the direct deposition of infectious seeds,^37^ which verified that ubiquitin reflected immune infiltration in alveoli, playing a role in representing the immune state.

Consensus clustering divided the ubiquitination profile of COVID‐19 PBMC into two subclusters, showing apparent differential traits in molecular function and clinical prognosis. Ubiquitination stratification was a favourable differentiator for COVID‐19 patients, with significant differences in clinical inflammatory indicators, mechanical ventilation probability, and ICU occupancy rates. The high ubiquitination of COVID‐19 patients revealed a favourable prognosis and depressed inflammatory states. CRP, D‐dimer, and ferritin have been used to evaluate a clinical inflammatory syndrome.^28^, ^38^ These indicators decreased in cluster 1 but increased in cluster 2; the higher ubiquitin cluster presented lower inflammation. From GO pathway enrichment annotation, upregulated genes with high ubiquitination groups mainly participated in the T cell‐associated immune response. There is growing evidence to support a potential role for T cell immunity in preventing initial infection and limiting the extent of disease after infection.^39^, ^40^, ^41^ T cell immunity may be essential for viral clearance to control SARS‐CoV‐2 infection.^42^ Upregulated genes of low ubiquitination were associated with negative cytokine‐mediated signalling regulation and positive neutrophil mediation regulation. Elevated neutrophils were concentrated in severe COVID‐19 patients, which could be attributed to the formation of neutrophil extracellular traps.^43^

Additionally, it has been reported that proteins escalate from healthy to mildly ill patients but decrease sharply from moderately to severely ill COVID‐19 patients.^44^ As a method of protein degradation ligating the proteasome, the ubiquitination level also demonstrated that mild symptoms were associated with high ubiquitin gene expression, and a low ubiquitin cluster was associated with poor prognosis. The results showed that ubiquitin transformation represented disease severity change. Hence, differences in inflammation and prognosis were due to distinct response patterns of ubiquitin subclusters.

Ubiquitination significantly interacts with immune responses and infiltration in inflammatory disorders,^45^, ^46^ and no exception for COVID‐19.^47^ The disparity of COVID‐19 patients' ubiquitin clusters demonstrated a divergent immune landscape in blood lymphocytes. The high ubiquitin cluster illustrated higher infiltrations of T cells, B cells, and DCs, whereas the depression of ubiquitination correlated with the elevation of basophils, neutrophils, platelets, epithelial cells, and endothelial cells. Accordingly, high ubiquitin patients reveal activation of the acquired immune response, suggesting a protective role of ubiquitination. Conversely, escalating inflammatory and coagulation dysfunction was consistent with the diminished ubiquitin pathway, which probably resulted in an undesirable prognosis consistent with previous reports. Compared with healthy controls, neutrophils increase, but DC and B cells decrease in acute respiratory distress syndrome patients with COVID‐19.^48^ It is striking that plasmacytoid dendritic cells, a major source of type I IFN, indicate the severity and that a decrease in T cells coincides with the deterioration of COVID‐19.^48^, ^49^ Neutrophil escalation and lymphocyte death are more common in severe than moderate cases, for example, T cell exhaustion forerunning irretrievable multiple organ dysfunction syndrome.^28^

Additionally, the proportion of neutrophils and eosinophils is amplified in severe COVID‐19 donors, consistent with the hypothesis that the neutrophil‐to‐lymphocyte ratio is an independent factor of severe disease.^50^ IGH and other immune molecules related to humoral responses were augmented in high ubiquitin clusters, while partial cytokines accumulated to contribute to the cytokine storm in lower clusters. The above results indicate that the high ubiquitin level and harmonious immune responses could promote beneficial protection and inhibit a damaging inflammatory response in COVID‐19. Therefore, clinical evaluations of this correlation and screening of predictors in ubiquitination have become urgent issues.

The ubiquitin consensus cluster showed the difference in severe disease in COVID‐19, which had important clinical value for screening critical patients. Due to its good discrimination ability, we screened out the differential genes to construct a patient risk model to evaluate the severity of the patient's disease. LASSO regression was used to set up the six‐molecule risk model based on whether mechanical ventilation was performed and the duration of mechanical ventilation. Superior performance was demonstrated by the mechanical ventilation probability curve and the area under the ROC curve. The grouping of models was significantly correlated with clinical indicators. Previous studies have reported that patients with severe COVID‐19 had relatively high CRP and higher SOFA and APACHE‐II scores,^51^, ^52^, ^53^ consistent with the model subgroups. There was a significant correlation between the model subgroup, clinical inflammatory indicators or mechanical ventilation, ICU occupancy rates, and other critical illness indices. In addition, the high‐risk group was significantly associated with low ubiquitination, consistent with our previous analysis. Therefore, it provides a new concept for COVID‐19 risk assessment and severity prediction.

The model consisted of six biomarkers, including three risk factors (CYSTM1, GLDN, and PCOLCE2) and three protective factors (SDSL, SEZ6L, and TNFRSF18). It has been reported that TNFRSF18 not only recognises follicular T cell subsets but also increases the ratio of T follicular helper cells to regulatory T follicular cells in and outside the germinal centre, which is a target for enhancing humoral immunity.^54^ TNFRF18 is also involved in the control of allergic lung inflammation.^55^ In contrast, PCOLCE2 can enhance collagen activity, and SARS‐CoV‐2 infection causes an increase in collagen 1 in organoids and promotes the activation of fibrotic signalling pathways.^56^ COVID‐19 patients showed increased diffusion of fibrinogen and collagen associated with platelet overreaction.^57^ The 6‐factor signature, thus, was promising in clinical application for prognostic evaluation of COVID‐19.

Although the significance of ubiquitinated molecules in COVID‐19 has been explored in our research from multiple perspectives, some inadequacies remained. First, our data was from public sources and lacked real‐world prospective studies. Second, according to the subcellular compartment, ubiquitin had a specific function and was mainly focused on the nuclear compartment (Figure 3E,F), which could result from promoting DNA damage and inhibiting DNA repair in SARS‐CoV‐2 infection, which needed further experiments to validate the role of ubiquitination in DNA synthesis. Third, ubiquitination only revealed the prognostic value of clinical patients with different modes of immune cell response and infiltration, of which further clarification of various cell types should be guaranteed in more detail through single‐cell sequencing or in situ cytometry studies.

In conclusion, we analysed and positively correlated the cross‐linking of ubiquitination and immune infiltration in COVID‐19 infection. Moreover, the ubiquitination stratification of peripheral blood was significantly correlated with the inflammatory response and prognostic indicators of patients, which was an important indicator for distinguishing COVID‐19 patients critically. Risk models and biomarker screening demonstrated superior performance in evaluating the prognosis of patients with COVID‐19, providing new ideas for clinical intervention and prognostic guidance.

## 4 METHOD

### 4.1 Data availability

The data on COVID‐19 ubiquitination originated from the Gene Expression Omnibus (https://www.ncbi.nlm.nih.gov/geo/) under accession numbers GSE162911, GSE147507, GSE184536, GSE182299, and GSE157103. GSE162911 included 9 COVID‐19 patients and 3 healthy controls, and a total of 401 standardised sequencing samples from eight morphologies were used to analyse. We selected count data of four lung autopsy samples from two COVID‐19 patients and two healthy negative controls in GSE147507, and counted data were converted to transcripts per million (TPM) data before analysis. In GSE184536, the RNA‐seq count data of 18 mock A549 cells and 17 A549 cells infected with SARS‐CoV‐2 were converted to TPM data and analysed by treatment for different periods (2, 4, 6, 9, 12, and 24 h). 120 autopsy samples of COVID‐19 patients were collected in GSE182299, which were resources from the bladder, heart, kidney, lung, lymph node, pancreas, and spleen. Additionally, the RNA‐seq normalised data in the blood leukocytes of 100 COVID‐19 and 26 non‐COVID‐19 patients were analysed in GSE 157103 to study the potential clinical value of ubiquitination.

The other data are available in the ProteomeXchange Consortium via the PRIDE41 partner repository with the dataset identifier PXD017710. Twelve Caco‐2 cells infected with SARS‐CoV‐2 were analysed in the normalised datasets after treatment for different periods (2, 6, 10, and 24 h). The data analysed in this study were from public databases and published articles, and the detailed sample information was summarised in Table S1.

### 4.2 Differential analysis of A549 cell ubiquitin genes between mock‐infected and SARS‐CoV‐2‐infected cells in different periods

Ubiquitination genes were integrated and summarised in Table S2. To profile the ubiquitination traits, we integrated 337 ubiquitin‐associated enzymes, including E1s, E2s, E3s, and DUEs. We calculated the mean expression of ubiquitin genes to represent the overall characteristics of ubiquitin better. Ubiquitin genes were screened from the transcriptome in A549 cells. According to the mock group infected with SARS‐CoV‐2, differential analysis was performed by the ‘limma’ package at different periods, including 2, 4, 6, 9, 12, and 24 h.

### 4.3 Principal component analysis

In PCA, ubiquitin genes were analysed by the ‘factoextra’ package to distinguish between the mock group and the infected SARS‐CoV‐2 group in A549 cells. Additionally, the PCA was grouped by mock and infecting SARS‐CoV‐2 groups of A549 cells in different periods (2, 4, 6, 9, 12, and 24 h). Meanwhile, ubiquitin proteins of Caco‐2 cells were subjected to PCA by grouping infecting periods of 2, 6, 10, and 24 h. The results showed a time‐dependent ubiquitination change after infection with SARS‐CoV‐2.

### 4.4 Estimation of immune cell infiltration fractions

To study the relationship between immune cell infiltration and ubiquitination of SARS‐CoV‐2, we used the ‘xCell’ package to analyse the normalised RNA‐seq data to demonstrate the immune and other cell characteristics. The ‘xCell’ package analysed 64 various cells, stroma scores, immune scores, and microenvironment scores by the ssGSEA algorithm.^58^ Hence, we analysed the traits of immune cells and others in the respiratory system and blood system infecting SARS‐CoV‐2.

### 4.5 Gene set variant analysis of ubiquitin genes in blood lymphocytes

To evaluate the biological function in the blood lymphocytes of non/COVID‐19 patients, we conducted GSVA enrichment of ubiquitin genes with the ‘GSVA’ package. GSVA estimated the variations in pathway activity over a sample population in an unsupervised manner.^59^ The biological progress (BP), cellular component (CC), and molecular function (MF) were annotated by ‘c5.go.bp.v7.4.symbols.gmt’, ‘c5.go.cc.v7.4.symbols.gmt’, and ‘c5.go.mf.v7.4.symbols.gmt’, respectively. They were downloaded from the MSigDB database for GSVA analysis. The significantly enriched pathways were filtered by an adjusted *p*‐value of <.05. The biological progress, cellular component, and molecular function pathways were subjected to differential analysis by the ‘limma’ package in the blood lymphocytes of non/COVID‐19 patients in GSE157103. The ubiquitin‐associated pathways were activated in COVID‐19 patients according to GSVA enrichment analysis.

### 4.6 Unsupervised clustering of ubiquitination in COVID‐19 patients

To study the impact of ubiquitination traits on COVID‐19 patients, a ubiquitin gene transcriptome including E1s, E2s, E3s, and deubiquitination (DUB) was subjected to clustering of its ‘euclidean’ distance by the ‘ConsensusClusterPlus’ package, which was an unsupervised clustering algorithm.^60^ When the *k* value equals 2, the effect of clustering was optimal, and ubiquitin genes clustered into two clusters, clusters 1 and 2. The ubiquitin genes were more highly expressed in cluster 1 than in cluster 2. PCA, grouping by ubiquitin clusters, was performed by comprehensive transcriptome data in blood lymphocytes by the ‘factoextra’ package. The proteasome and ubiquitin‐mediated proteolysis pathways were enriched by GSVA analysis annotating ‘c2.cp.kegg.v7.1.symbols.gmt’ downloaded from the MSigDB database. The highly expressed genes of clusters 1 and 2 were subjected to GO pathway enrichment analysis by the ‘clusterProfiler’ package in R.

### 4.7 Construction of the risk model

Differential analysis was conducted between clusters 1/2 and non/COVID‐19 to acquire 974 and 4292 genes, respectively. Intersecting the same 355 genes could somewhat reflect the prognosis of COVID‐19 patients. The patients were divided into two groups: the training cohort consisted of 60% of the patients (including 48 patients), while the test cohort consisted of 40% of the patients (including 32 patients). We constructed a model in the training patient cohort based on 355 genes using the ‘glmnet’ package by the LASSO, which constructs a first‐order penalty function to obtain a refined model. The final signatures were filtered by determining the appropriate *λ* value with 20‐fold cross‐validation and ‘deviance’ as the target parameter. The coefficients of the final signatures were used to calculate the risk score as follows:

risk score = ∑i[Coefficient(mRNAi) × Expression(mRNAi)].

The model was validated in the test cohort. The risk score was calculated as follows:

Risk score = CYSTM1* 0.126+ GLDN* 0.046+ PCOLCE2* 0.051+ SDSL*(−0.084) + SEZ6L*(−0.132) + TNFRSF18*(−0.185).

The time‐dependent area under the ROC was plotted with area under curve ROC (AUC) scores using the R package ‛survivalROC’ to evaluate the time probability of mechanical ventilation and the model's performance.

The functional protein interaction network of the six biomarker genes was predicted using the STRING database (https://string‐db.org/) and considering the interacting proteins based on an interaction score >0.40. Sixty‐seven molecular proteins met the screening criteria, and a protein interaction network map was constructed by using Cytoscape 3.6.1. These proteins were subjected to GO pathway enrichment analysis by the 'clusterProfiler' package in R.

### 4.8 Statistical analysis

Differences between the two groups were compared using the Wilcoxon sum‐rank test or the *t*‐test. Differences among three or more groups were compared using the Kruskal–Wallis test. The chi‐square test was used to test for correlation between the two variables. All statistical tests were two‐sided, and a *p*‐value of <.05 was considered statistically significant.

## CONFLICT OF INTEREST

The authors declare no conflict of interest.

## Supporting information

Supporting Information

Supporting Information

Supporting Information

Supporting Information

Supporting Information

Supporting Information

Supporting Information

Supporting Information

Supporting Information

## References

[1] Davies NG , Abbott S , Barnard RC , et al. Estimated transmissibility and impact of SARS‐CoV‐2 lineage B.1.1.7 in England. Science. 2021;372(6538):149. 10.1126/science.abg3055 PMC8128288 33658326

[2] Tegally H , Wilkinson E , Giovanetti M , et al. Detection of a SARS‐CoV‐2 variant of concern in South Africa. Nature. 2021;592(7854):438. 33690265 10.1038/s41586-021-03402-9

[3] Faria NR , Mellan TA , Whittaker C , et al. Genomics and epidemiology of the P.1 SARS‐CoV‐2 lineage in Manaus, Brazil. Science. 2021;372(6544):815. 33853970 10.1126/science.abh2644 PMC8139423

[4] Allen H , Vusirikala A , Flannagan J , et al. Household transmission of COVID‐19 cases associated with SARS‐CoV‐2 delta variant (B.1.617.2): national case‐control study. Lancet Reg Health Eur. 2022;12:100252. 34729548 10.1016/j.lanepe.2021.100252 PMC8552812

[5] Wang P , Casner RG , Nair MS , et al. Increased resistance of SARS‐CoV‐2 variant P.1 to antibody neutralization. Cell Host Microbe. 2021;29(5):747‐751. 33887205 10.1016/j.chom.2021.04.007 PMC8053237

[6] Zhu N , Zhang DY , Wang WL , et al. A novel coronavirus from patients with pneumonia in China, 2019. New Engl J Med. 2020;382(8):727‐733. 31978945 10.1056/NEJMoa2001017 PMC7092803

[7] Tan BK , Mainbourg S , Friggeri A , et al. Arterial and venous thromboembolism in COVID‐19: a study‐level meta‐analysis. Thorax. 2021;76(10):970‐979. 33622981 10.1136/thoraxjnl-2020-215383

[8] Puelles VG , Lutgehetmann M , Lindenmeyer MT , et al. Multiorgan and renal tropism of SARS‐CoV‐2. N Engl J Med. 2020;383(6):590‐592. 32402155 10.1056/NEJMc2011400 PMC7240771

[9] Petersen EL , Gossling A , Adam G , et al. Multi‐organ assessment in mainly non‐hospitalized individuals after SARS‐CoV‐2 infection: the Hamburg City Health Study COVID programme. Eur Heart J. 2022;43(11):1124‐1137. 34999762 10.1093/eurheartj/ehab914 PMC8755397

[10] Jaffray EG , Hay RT . Detection of modification by ubiquitin‐like proteins. Methods. 2006;38(1):35‐38. 16343933 10.1016/j.ymeth.2005.07.020

[11] Isaacson MK , Ploegh HL . Ubiquitination, ubiquitin‐like modifiers, and deubiquitination in viral infection. Cell Host Microbe. 2009;5(6):559‐570. 19527883 10.1016/j.chom.2009.05.012 PMC7103382

[12] Stukalov A , Girault V , Grass V , et al. Multilevel proteomics reveals host perturbations by SARS‐CoV‐2 and SARS‐CoV. Nature. 2021;594(7862):246‐252. 33845483 10.1038/s41586-021-03493-4

[13] Mahon C , Krogan NJ , Craik CS . Pick E: cullin E3 ligases and their rewiring by viral factors. Biomolecules. 2014;4(4):897‐930. 25314029 10.3390/biom4040897 PMC4279162

[14] Timms RT , Zhang ZQ , Rhee DY , Harper JW , Koren I , Elledge SJ . A glycine‐specific N‐degron pathway mediates the quality control of protein N‐myristoylation. Science. 2019;365(6448):45. 10.1126/science.aaw4912 PMC7090375 31273098

[15] Gordon DE , Jang GM , Bouhaddou M , et al. A SARS‐CoV‐2 protein interaction map reveals targets for drug repurposing. Nature. 2020;583(7816):459. 32353859 10.1038/s41586-020-2286-9 PMC7431030

[16] Savellini GG , Anichini G , Gandolfo C , Cusi MG . SARS‐CoV‐2 N protein targets TRIM25‐mediated RIG‐I activation to suppress innate immunity. Viruses‐Basel. 2021;13(8):1439. 10.3390/v13081439 PMC8402637 34452305

[17] Novelli G , Liu J , Biancolella M , et al. Inhibition of HECT E3 ligases as potential therapy for COVID‐19. Cell Death Dis. 2021;12(4):310. 10.1038/s41419-021-03513-1 PMC7987752 33762578

[18] Wang S , Yao X , Ma S , Ping Y , et al. A single‐cell transcriptomic landscape of the lungs of patients with COVID‐19. Nat Cell Biol. 2021;23(12):1314‐1328. 34876692 10.1038/s41556-021-00796-6 PMC8650955

[19] Elia AE , Boardman AP , Wang DC , et al. Quantitative proteomic atlas of ubiquitination and acetylation in the DNA damage response. Mol Cell. 2015;59(5):867‐881. 26051181 10.1016/j.molcel.2015.05.006 PMC4560960

[20] Liu J , Han C , Xie B , Wu Y , et al. Rhbdd3 controls autoimmunity by suppressing the production of IL‐6 by dendritic cells via K27‐linked ubiquitination of the regulator NEMO. Nat Immunol. 2014;15(7):612‐622. 24859449 10.1038/ni.2898

[21] Gatti M , Pinato S , Maiolica A , et al. RNF168 promotes noncanonical K27 ubiquitination to signal DNA damage. Cell Rep. 2015;10(2):226‐238. 25578731 10.1016/j.celrep.2014.12.021

[22] Yin Q , Han T , Fang B , Zhang GL , et al. K27‐linked ubiquitination of BRAF by ITCH engages cytokine response to maintain MEK‐ERK signaling. Nat Commun. 2019;10(1):1870. 31015455 10.1038/s41467-019-09844-0 PMC6478693

[23] Gao P , Ma X , Yuan M , et al. E3 ligase Nedd4l promotes antiviral innate immunity by catalyzing K29‐linked cysteine ubiquitination of TRAF3. Nat Commun. 2021;12(1):1194. 33608556 10.1038/s41467-021-21456-1 PMC7895832

[24] Fouad S , Wells OS , Hill MA , D'Angiolella V . Cullin ring ubiquitin ligases (CRLs) in cancer: responses to ionizing radiation (IR) treatment. Front Physiol. 2019;10:1144. 31632280 10.3389/fphys.2019.01144 PMC6781834

[25] Ulrich HD , Walden H . Ubiquitin signalling in DNA replication and repair. Nat Rev Mol Cell Bio. 2010;11(7):479‐489. 20551964 10.1038/nrm2921

[26] Breckel CA , Hochstrasser M . Ubiquitin ligase redundancy and nuclear‐cytoplasmic localization in yeast protein quality control. Biomolecules. 2021;11(12):1821. 34944465 10.3390/biom11121821 PMC8698790

[27] Overmyer KA , Shishkova E , Miller IJ , et al. Large‐scale multi‐omic analysis of COVID‐19 severity. Cell Syst. 2021;12(1):23. 33096026 10.1016/j.cels.2020.10.003 PMC7543711

[28] Chen G , Wu D , Guo W , et al. Clinical and immunological features of severe and moderate coronavirus disease 2019. J Clin Invest. 2020;130(5):2620‐2629. 32217835 10.1172/JCI137244 PMC7190990

[29] Kidd P . Th1/Th2 balance: the hypothesis, its limitations, and implications for health and disease. Altern Med Rev. 2003;8(3):223‐246. 12946237

[30] Gil‐Etayo FJ , Suarez‐Fernandez P , Cabrera‐Marante O , et al. T‐helper cell subset response is a determining factor in COVID‐19 progression. Front Cell Infect Mi. 2021;11:624483. 10.3389/fcimb.2021.624483 PMC7952877 33718270

[31] Song CY , Xu J , He JQ , Lu YQ . Immune dysfunction following COVID‐19, especially in severe patients. Sci Rep. 2020;10(1):15838. 32985562 10.1038/s41598-020-72718-9 PMC7522270

[32] Wang J , Jiang MM , Chen X , Montaner LJ . Cytokine storm and leukocyte changes in mild versus severe SARS‐CoV‐2 infection: review of 3939 COVID‐19 patients in China and emerging pathogenesis and therapy concepts. J Leukocyte Biol. 2020;108(1):17‐41. 32534467 10.1002/JLB.3COVR0520-272R PMC7323250

[33] Pastorek M , Dubrava M , Celec P . On the origin of neutrophil extracellular traps in COVID‐19. Front Immunol. 2022;13:821007. 35359960 10.3389/fimmu.2022.821007 PMC8961727

[34] Grossegesse M , Doellinger J , Fritsch A , et al. Global ubiquitination analysis reveals extensive modification and proteasomal degradation of cowpox virus proteins, but preservation of viral cores. Sci Rep. 2018;8(1):1807. 29379051 10.1038/s41598-018-20130-9 PMC5788924

[35] Zhang H , Zheng H , Zhu J , et al. Ubiquitin‐modified proteome of SARS‐CoV‐2‐Infected host cells reveals insights into virus‐host interaction and pathogenesis. J Proteome Res. 2021;20(5):2224‐2239. 33666082 10.1021/acs.jproteome.0c00758

[36] Gao WY , Wang LL , Ju XH , et al. The deubiquitinase USP29 promotes SARS‐CoV‐2 virulence by preventing proteasome degradation of ORF9b. Mbio. 2022;13(3):e0130022. 35638730 10.1128/mbio.01300-22 PMC9239186

[37] Chen A , Wessler T , Daftari K , et al. Modeling insights into SARS‐CoV‐2 respiratory tract infections prior to immune protection. Biophys J. 2022;121(9):1619‐1631. 35378080 10.1016/j.bpj.2022.04.003 PMC8975607

[38] Mathew D , Giles JR , Baxter AE , Oldridge DA , et al. Deep immune profiling of COVID‐19 patients reveals distinct immunotypes with therapeutic implications. Science. 2020;369(6508):1209. 10.1126/science.abc8511 PMC7402624 32669297

[39] Moss P . The T cell immune response against SARS‐CoV‐2. Nat Immunol. 2022;23(2):186‐193. 35105982 10.1038/s41590-021-01122-w

[40] Jogdand GM , Mohanty S , Devadas S . Regulators of Tfh cell differentiation. Front Immunol. 2016;7:520. 27933060 10.3389/fimmu.2016.00520 PMC5120123

[41] Tang R , Langdon WY , Zhang J . Regulation of immune responses by E3 ubiquitin ligase Cbl‐b. Cellular Immunology. 2019;340:103878. 30442330 10.1016/j.cellimm.2018.11.002 PMC6504630

[42] Zeng C , Evans JP , King T . SARS‐CoV‐2 spreads through cell‐to‐cell transmission. P Natl Acad Sci USA. 2022;119(1):e2111400119. 10.1073/pnas.2111400119 PMC8740724 34937699

[43] Pastorek M , Dubrava M , Celec P . On the origin of neutrophil extracellular traps in COVID‐19. Front Immunol. 2022;13:821007. 35359960 10.3389/fimmu.2022.821007 PMC8961727

[44] Su YP , Chen D , Yuan D , et al. Multi‐omics resolves a sharp disease‐state shift between mild and moderate COVID‐19. Cell. 2020;183(6):1479. 33171100 10.1016/j.cell.2020.10.037 PMC7598382

[45] Tang R , Langdon WY , Zhang J . Regulation of immune responses by E3 ubiquitin ligase Cbl‐b. Cell Immunol. 2019;340:103878. 30442330 10.1016/j.cellimm.2018.11.002 PMC6504630

[46] Yu H , Lin L , Zhang Z , Zhang H , Hu H . Targeting NF‐kappaB pathway for the therapy of diseases: mechanism and clinical study. Signal Transduct Target Ther. 2020;5(1):209. 32958760 10.1038/s41392-020-00312-6 PMC7506548

[47] Zhao Y , Sui L , Wu P , et al. A dual‐role of SARS‐CoV‐2 nucleocapsid protein in regulating innate immune response. Signal Transduct Target Ther. 2021;6(1):331. 34471099 10.1038/s41392-021-00742-w PMC8409078

[48] Wilk AJ , Rustagi A , Zhao NQ , et al. A single‐cell atlas of the peripheral immune response in patients with severe COVID‐19. Nat Med. 2020;26(7):1070‐1076. 32514174 10.1038/s41591-020-0944-y PMC7382903

[49] Laing AG , Lorenc A , del Molino Del Barrio I , et al. Author correction: a dynamic COVID‐19 immune signature includes associations with poor prognosis. Nat Med. 2020;26(12):1951. 10.1038/s41591-020-01186-5 PMC7694581 33247289

[50] Kuri‐Cervantes L , Pampena MB , Meng WZ , et al. Comprehensive mapping of immune perturbations associated with severe COVID‐19. Science Immunology. 2020;5(49):eabd7114. 32669287 10.1126/sciimmunol.abd7114 PMC7402634

[51] Smilowitz NR , Kunichoff D , Garshick M , et al. C‐reactive protein and clinical outcomes in patients with COVID‐19. Eur Heart J. 2021;42(23):2270‐2279. 33448289 10.1093/eurheartj/ehaa1103 PMC7928982

[52] Bauer A , Schreinlechner M , Sappler N , et al. Discontinuation versus continuation of renin‐angiotensin‐system inhibitors in COVID‐19 (ACEI‐COVID): a prospective, parallel group, randomised, controlled, open‐label trial. Lancet Respir Med. 2021;9(8):863‐872. 34126053 10.1016/S2213-2600(21)00214-9 PMC8195495

[53] Liu YA , Xia P , Cao W , et al. Divergence between serum creatine and cystatin C in estimating glomerular filtration rate of critically ill COVID‐19 patients. Renal Failure. 2021;43(1):1104‐1114. 34238117 10.1080/0886022X.2021.1948428 PMC8274508

[54] Oja AE , Brasser G , Slot E , et al. GITR shapes humoral immunity by controlling the balance between follicular T helper cells and regulatory T follicular cells. Immunol Lett. 2020;222:73‐79. 32259529 10.1016/j.imlet.2020.03.008

[55] Nagashima H , Okuyama Y , Fujita T , et al. GITR cosignal in ILC2s controls allergic lung inflammation. J Allergy Clin Immun. 2018;141(5):1939. 29427641 10.1016/j.jaci.2018.01.028

[56] Jansen J , Reimer KC , Nagai JS , et al. SARS‐CoV‐2 infects the human kidney and drives fibrosis in kidney organoids. Cell Stem Cell. 2022;29(2):217. 35032430 10.1016/j.stem.2021.12.010 PMC8709832

[57] Manne BK , Denorme F , Middleton EA , et al. Platelet gene expression and function in patients with COVID‐19. Blood. 2020;136(11):1317‐1329. 32573711 10.1182/blood.2020007214 PMC7483430

[58] Aran D , Hu ZC , Butte AJ . xCell: digitally portraying the tissue cellular heterogeneity landscape. Genome Biol. 2017;18(1):220. 29141660 10.1186/s13059-017-1349-1 PMC5688663

[59] Hanzelmann S , Castelo R , Guinney J . GSVA: gene set variation analysis for microarray and RNA‐Seq data. BMC Bioinformatics. 2013;14:7. 23323831 10.1186/1471-2105-14-7 PMC3618321

[60] Wilkerson MD , Hayes DN . ConsensusClusterPlus: a class discovery tool with confidence assessments and item tracking. Bioinformatics. 2010;26(12):1572‐1573. 20427518 10.1093/bioinformatics/btq170 PMC2881355

