# Supplementary figures and images for: Elevated ubiquitination contributes to protective immunity against severe SARS‐CoV‐2 infection
Source: Clin Transl Med. 2022 Nov 29;12(12):e1103. doi: 10.1002/ctm2.1103 (PMC9708907; doi:10.1002/ctm2.1103)

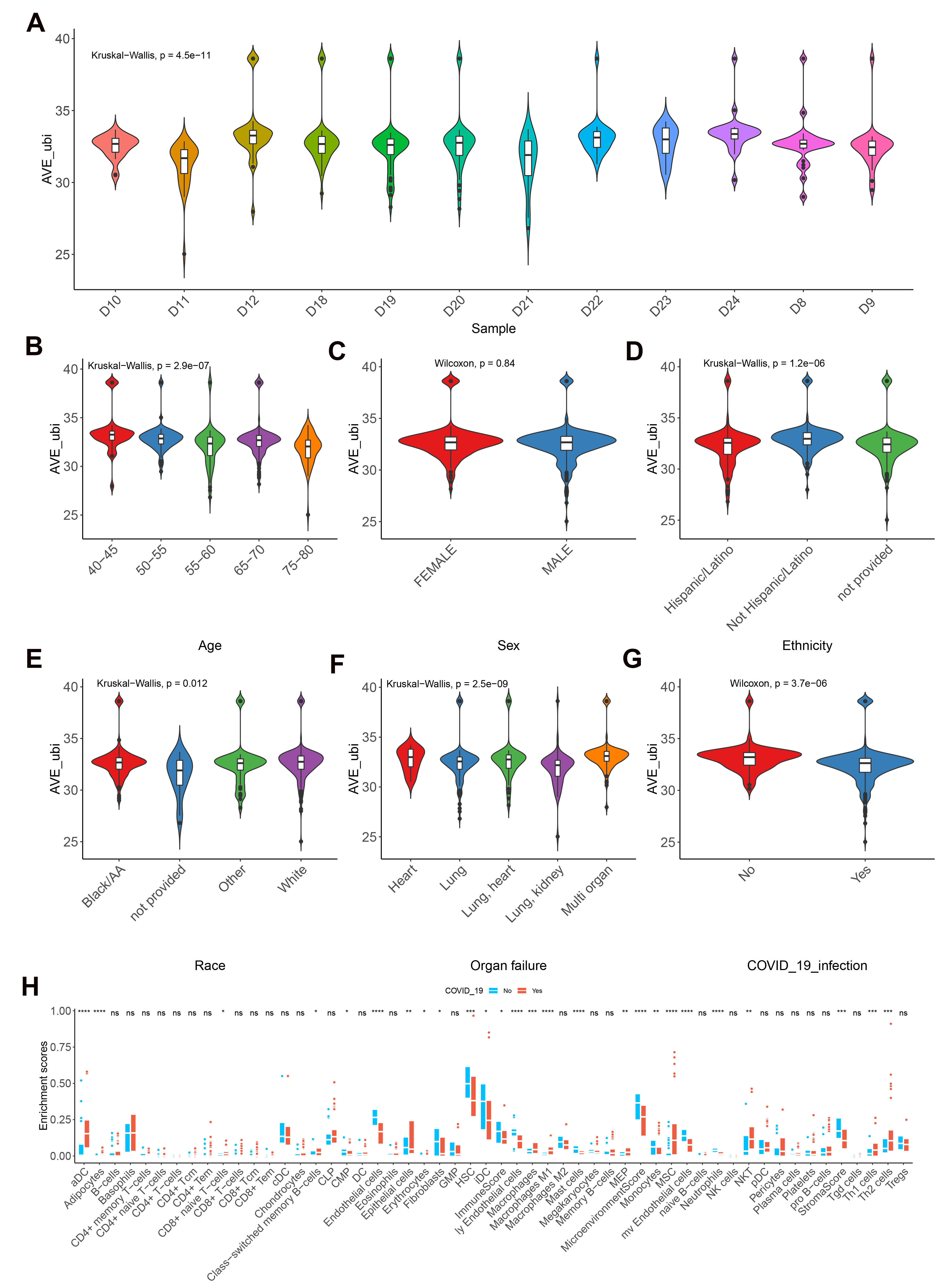

Supplement: Supplementary file 1 — Supporting Information [file CTM2-12-0-s004.jpg]

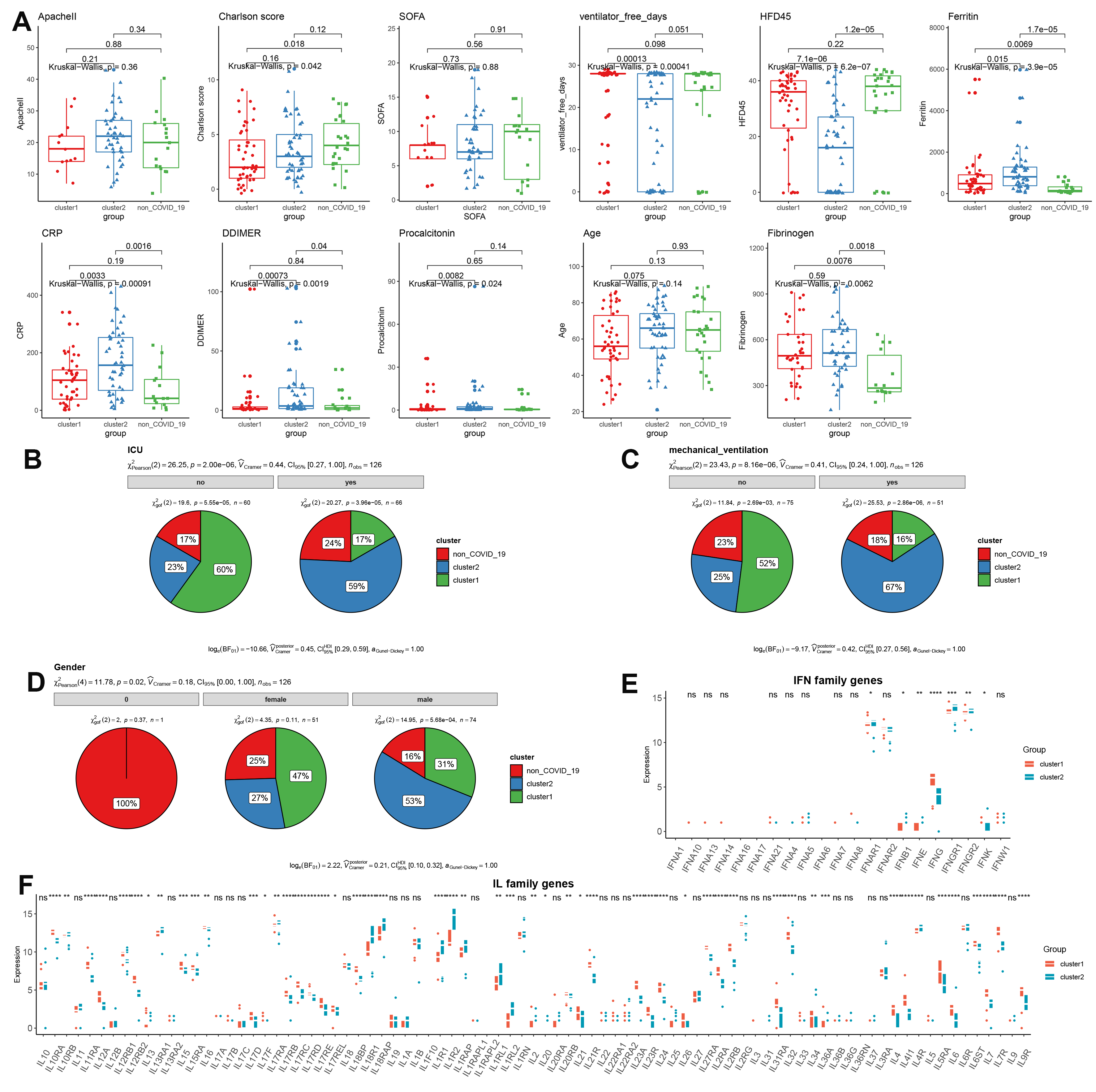

Supplement: Supplementary file 2 — Supporting Information [file CTM2-12-0-s009.png]

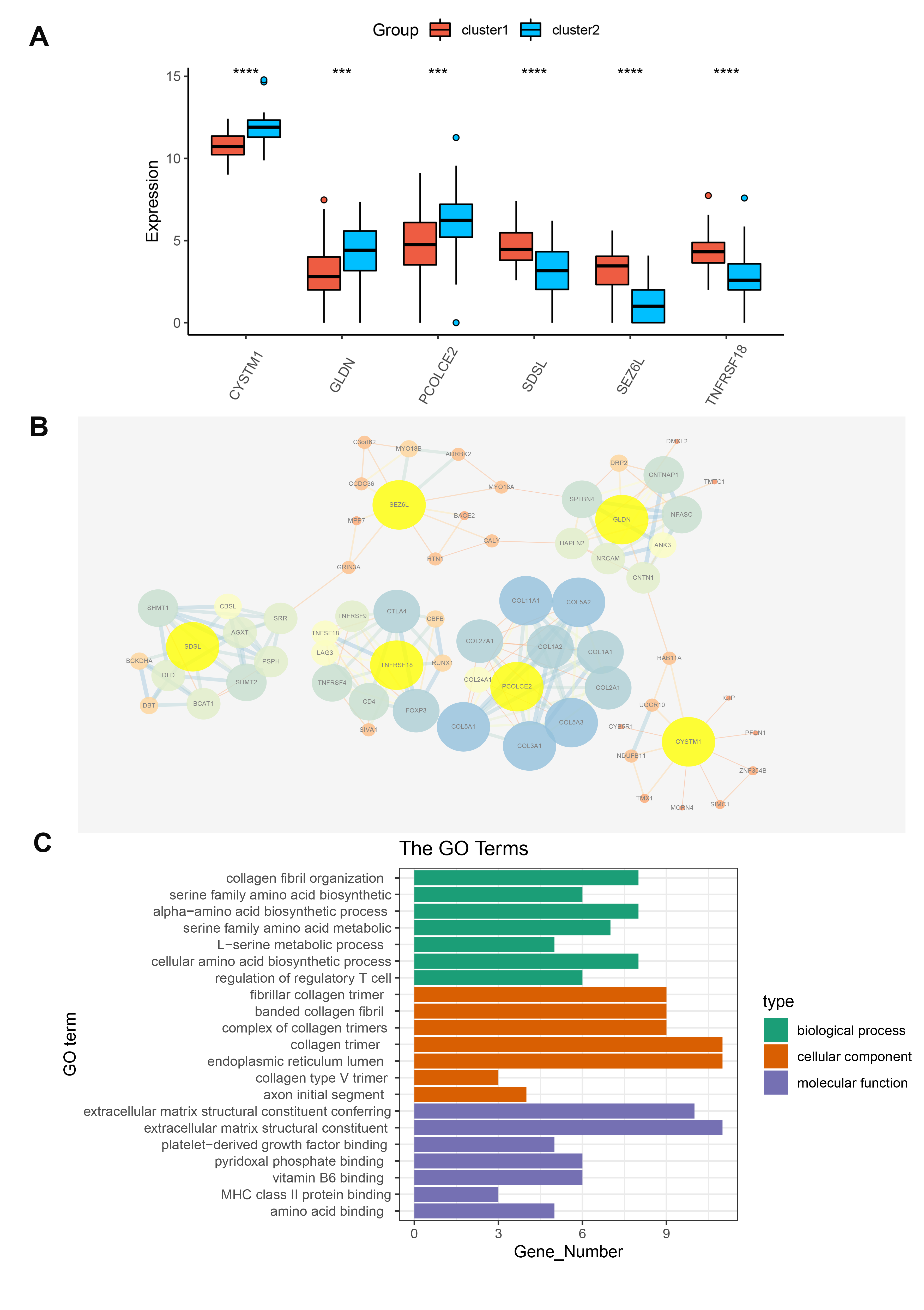

Supplement: Supplementary file 3 — Supporting Information [file CTM2-12-0-s008.png]
